# The role of bacteria in wastewater treatment and the impact of treated wastewater on riverine bacterial ecosystems

**DOI:** 10.1371/journal.pone.0346342

**Published:** 2026-04-15

**Authors:** Akifumi Nishida, Mayuko Nakagawa, Masayuki Yamamura

**Affiliations:** 1 Department of Molecular Microbiology, Tokyo University of Agriculture, Tokyo, Japan; 2 Department of Earth and Planetary Sciences, Institute of Science Tokyo, Tokyo, Japan; 3 Department of Computer Science, Institute of Science Tokyo, Kanagawa, Japan; Nanjing University of Science and Technology, CHINA

## Abstract

Spatiotemporal analysis of bacterial communities in wastewater can explain the role of bacteria in removing organic matter, phosphorus, and nitrogen as well as the impact of wastewater treatment on riverine ecosystems. This study investigated the bacterial dynamics within an anaerobic–anoxic–oxic (A_2_O) wastewater treatment plant (WWTP), comprising anaerobic, anoxic, and aerobic tanks, in Tokyo and its impact on the receiving Tama River. 16S rRNA gene analysis and ion chromatography were used to monitor bacterial composition and nutrient concentrations, respectively, to assess the effectiveness of nutrient removal across seasonal temperature variations, and the influence of treated wastewater on riverine bacterial communities. The A_2_O process effectively removed nutrients, but the nitrification efficiency was affected by temperature, with decreased temperatures correlating with reduced *Nitrospira* abundance, while nitrate concentrations increased due to higher influent ammonium loads. The WWTP bacterial community exhibited a polarized structure with a dominant core community that was stable over time and across tanks. The abundance of bacterial DNA introduced into the river via treated wastewater decreased downstream, indicating the spatial attenuation of the wastewater treatment impact on the riverine ecosystem. This study demonstrated the temperature sensitivity of WWTP processes, and the transient impact of treated wastewater discharge on river bacterial communities, thereby emphasizing the importance of understanding these dynamics for effective environmental conservation.

## Introduction

Wastewater treatment plants (WWTPs) utilize bacteria to remove nutrients from wastewater and help maintain the ecosystems of rivers and oceans where the treated wastewater is discharged. Phosphorus and nitrogen are essential for the growth of life, and when wastewater, containing these nutrients, is released into the environment without treatment, it causes environmental stress [1–4]. Water quality deteriorates at the treated wastewater discharge point, causing the water temperature and nutrient concentrations to increase and dissolved oxygen to decrease. Many ecological studies have reported that this change has a negative effect on aquatic invertebrates and fish [5–9].

For example, the anaerobic–anoxic–oxic (A_2_O) process, which is common in WWTPs, can remove organic matter, nitrogen, and phosphorus from wastewater. The A_2_O process comprises three consecutive tanks—anaerobic, anoxic, and aerobic (oxic). Phosphorus-accumulating organisms (PAOs) release phosphorus in the anaerobic environment, absorb more phosphorus than they release in the aerobic environment, and finally, phosphorus is removed as sludge containing the PAOs. In the aerobic tank, nitrification occurs by autotrophic bacteria, and ammonia (NH_3_) is first oxidized to nitrite (NO_2_^-^) by ammonia-oxidizing bacteria, and then nitrite (NO_2_^-^) is oxidized to nitrate (NO_3_^-^) by nitrite-oxidizing bacteria. Internal recycling occurs from the aerobic to the anoxic tank, and nitrite and/or nitrate are used as electron acceptors. Finally, nitrate is reduced to nitrogen gas, although conditions such as temperature, dissolved oxygen, pH, and supplied ammonium concentration can lead to the generation of the greenhouse gas nitrous oxide [10–12]. It is important to analyze how the chemical composition of wastewater is altered by bacteria during the A_2_O process so as to control the nutrients in the wastewater.

Spatiotemporal measurements of bacteria and chemical compositions in wastewater treatment processes provide insights into how the process controls bacteria-mediated nutrient conditions. Furthermore, it is important to analyze the bacterial communities, both in the wastewater treatment process and the receiving river for understanding the impact of the discharged treated‑wastewater on the river ecosystem. While most studies on the effects of treated wastewater on rivers have focused on aquatic invertebrates and fish, only some have examined the effects on bacteria that play a fundamental role in nutrient cycling. At WWTPs in Chicago, USA, an increase in inorganic nutrients and a decrease in bacterial community diversity were observed when treated wastewater was discharged into rivers [13,14]. Antimicrobial resistance genes have also been detected in receiving rivers, raising concerns about the effect of the resistome (set of all antibiotic resistance genes) on bacterial communities [15]. In the Tama River in Japan, the discharge of treated wastewater caused major changes in the chemical composition of the river and deterministic changes in the structure of the bacterial community [16,17]. Given the significant impact of treated wastewater on riverine ecosystems demonstrated in these studies, clarifying the spatial dynamics of bacteria introduced via wastewater along the river is crucial for assessing potential ecological risks, such as the dispersal of antibiotic-resistant bacteria.

In this study, we hypothesized that seasonal temperature variations act as a deterministic factor reducing the abundance of key nitrifiers, such as *Nitrospira*, within the A_2_O process, thereby altering the bacterial composition discharged into the environment. Furthermore, we hypothesized that while treated wastewater discharge introduces distinct bacterial taxa into the receiving river, their persistence would be spatially limited due to physical dispersion and environmental removal processes. To test these hypotheses, we monitored the bacterial communities in the anaerobic, anoxic, and aerobic tanks of an A_2_O process in Tokyo and the receiving Tama River from summer through winter using 16S rRNA gene analysis alongside chemical composition analysis using ion chromatography.

## Materials and methods

### Ethics statement

This study was conducted with the formal approval of the Bureau of Sewerage, Tokyo Metropolitan Government. Access to the wastewater treatment plant and the use of samples were approved by the Director of the Environmental Management Section, Facilities Management Division, on August 1, 2018. The publication of the research results was officially authorized by the Director of the Facilities Management Section, Technology Department, Regional Sewerage Office, on March 17, 2025. As this study analyzed bacterial communities in wastewater and river water and did not involve human participants or vertebrate animals, specific informed consent from individuals was not required.

### Sample site and sample collection

**Fig 1:**
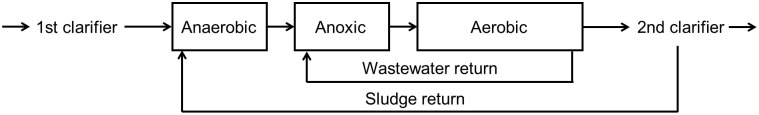
Anaerobic-Anoxic-Aerobic (A_2_O) treatment process. A portion of wastewater is returned from the aerobic tank to the anoxic tank, and the sludge from the 2nd clarifier is returned to the anaerobic tank.

The urban WWTP in Tokyo, using the A_2_O treatment process, discharges treated water into the Tama River (Fig 1). The A_2_O treatment process consisted of an anaerobic tank, an anoxic tank, and an aerobic tank; the capacity and residence time of each tank are shown in S1 Table. Operational parameters of the A_2_O process were provided by the Tokyo Metropolitan Government Bureau of Sewerage. These parameters were the oxidation-reduction potential (ORP) in the anaerobic and anoxic tanks, mixed liquor suspended solids (MLSS), dissolved oxygen (DO), and influent temperature (monitored monthly) (S2 Table). Samples of wastewater (300 mL) were collected in sterile polypropylene bottles (AS ONE, Osaka, Japan) from the outlet at the end of each tank once every two weeks from August 2018 to February 2019. The samples were stored on ice and DNA was isolated within 3 h. River water samples were collected from the Tama River at site S3 (treated wastewater released), site S1 (1 km upstream of site S3), site S4 (2 km downstream of site S3), and site S2 (treated wastewater). The specific geographic coordinates of all sampling sites are provided in S3 Table. The sampling amount and storage method were the same as when the wastewater was collected.

### DNA isolation, polymerase chain reaction (PCR) amplification, sequencing, and data availability

Genomic DNA was isolated from the wastewater and river water samples using a DNeasy PowerWater Sterivex DNA Isolation Kit (Qiagen, Hilden, Germany). PCR amplification of the 16S rRNA gene V3 and V4 variable regions and MiSeq sequencing (Illumina, San Diego, CA, USA) using the MiSeq Reagent Kit v3 (600 cycles) (Illumina, San Diego, CA, USA) were performed as previously described [18]. Sequencing data were deposited in the National Center for Biotechnology Information (Accession: PRJNA1235227).

### Microbiome analysis based on 16S rRNA gene sequences

The basic method used for microbiome analysis has been described previously [17]. The QIIME2 Docker software (qiime2–2019.10) was used to analyze the 16S rRNA gene sequences [19]. A total of 300 nucleotide (nt) paired-end reads were trimmed using the *qiime dada2 denoise-paired* command (31–299 nt forward and 17–278 nt reverse). The reads were taxonomically classified with 99% amplicon sequence variant (ASV) data using SILVA 132 [20] and the *qiime feature-classifier extract-reads* command (CTACGGGGGGCAGCAG for forward and GGACTACCGGGGTATCT for reverse). α-diversity was calculated using QIIME2 with a sampling depth of 10,000 reads. The weighted UniFrac distance was calculated using QIIME2 for Principal coordinate analysis (PCoA). PCoA was performed with environmental variables using the *envfit* function of the R vegan package (version 2.6–10) [21,22], and environmental variable vectors significantly correlated with ordination axes (*p* < 0.05) were displayed.

To evaluate the natural spatial variation of bacterial communities in the Tama River, we utilized 16S rRNA gene sequencing data from two upstream sites (upstream 1 and upstream 2) collected on August 31, 2018, as described in our previous study [17]. These sites are located upstream of the study area and are separated by approximately 8.5 km. The similarity of the bacterial communities between these two reference sites was analyzed to establish a baseline for natural riverine heterogeneity.

### Statistical analysis

The normality of the data distribution was assessed using the Shapiro-Wilk test. Since the bacterial relative abundance data did not follow a normal distribution, non-parametric tests were employed.

To compare bacterial communities across river sites (S1–S4) while accounting for temporal variability (sampling dates), the Friedman test (a non-parametric alternative to repeated measures ANOVA) was performed using the friedmanchisquare function from the SciPy library (version 1.14.1) in Python [23]. Post-hoc pairwise comparisons were conducted using the Nemenyi test with the *posthoc_nemenyi_friedman* function from the scikit-posthocs library (version 0.11.3) [24].

For beta-diversity analysis, the Weighted UniFrac distance was selected as the similarity metric because it accounts for both the phylogenetic relatedness of bacterial sequences and their relative abundances. Differences in community structures between tanks were tested using Permutational Multivariate Analysis of Variance (PERMANOVA) based on Weighted UniFrac distances. PERMANOVA was conducted using the *adonis* function in the R vegan package (version 2.6–10) with 999 permutations.

To assess relationships between ion concentrations and temperature, Spearman’s rank correlation analysis was performed using the *spearmanr* function from the SciPy library (version 1.6.3) in Python. For all statistical tests, a p-value of < 0.05 was considered statistically significant.

### Ion chromatography analysis in A_2_O treatment system

Water samples for ion chromatography analysis were collected using 50 mL syringes and filtered with membrane syringe filters of 0.20 μm pore size (DISMIC–25AS; Advantec Toyo Kaisha, Tokyo, Japan) as previously described [17]. Anion concentrations were measured by ion chromatography using a Shimadzu Ion Chromatograph (Shimadzu, Kyoto, Japan) equipped with a Shodex SI-90 4E anion column (Showa Denko, Tokyo, Japan). A Shim-pack IC-C4 (Shimadzu, Kyoto, Japan) without a cation suppressor was used.

## Results and discussion

### Bacterial and chemical composition in A_2_O treatment process

**Fig 2:**
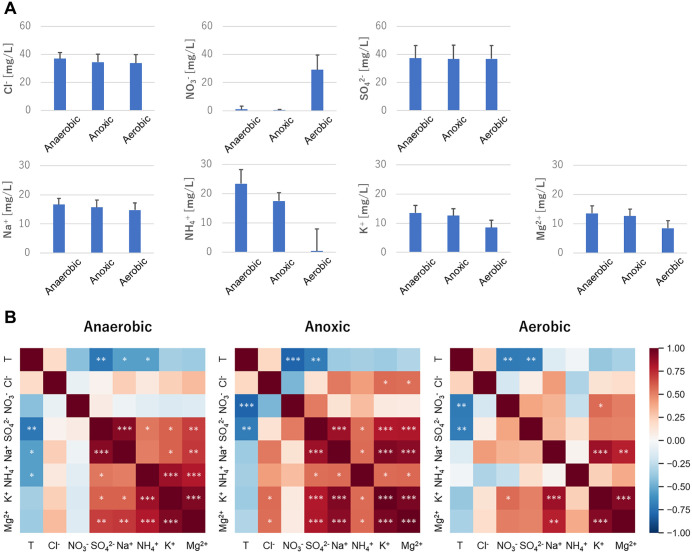
Chemical composition and correlations in A_2_O treatment process. **(A)** Ion concentrations in anaerobic, anoxic, and aerobic tanks. **(B)** Correlations between ion concentrations and temperature **(T)**. * indicates *p* < 0.05, ** indicates *p* < 0.01, and *** indicates *p* < 0.001.

**Fig 3:**
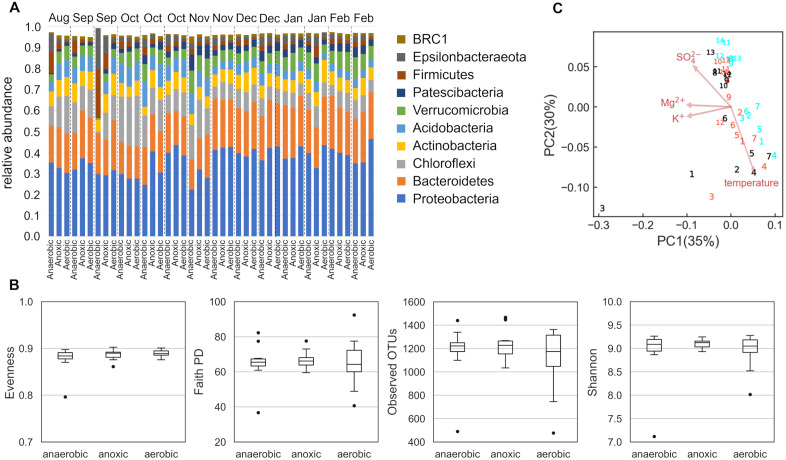
Bacterial composition in A_2_O treatment process. (A) bacterial composition at phylum level with top 10 bacteria. **(B)** Comparison of α-diversity indices (Evenness, Faith’s PD, Observed OTUs, Shannon) between tanks. **(C)** PCoA based on weighted UniFrac distance of microbiota. The numbers on the markers indicate the sampling period, with 1 representing summer and the numbers progressing towards 14 representing winter. The colors of the markers indicate the sample source: black for the anaerobic tank, orange for the anoxic tank, and cyan for the aerobic tank.

Analysis of the spatial chemical composition of wastewater from the anaerobic, anoxic, and aerobic tanks in the A_2_O treatment process revealed significant alterations in nitrogen compounds (Fig 2A, S1 Dataset). Specifically, the process achieved a high ammonium removal efficiency of 98.4 ± 5.8% (mean ± SD). This effective nitrification was characterized by a sharp decrease in ammonium (NH₄⁺) and a concurrent increase in nitrate (NO₃⁻) within the aerobic tank. Bacterial metabolic activities contribute to these chemical changes, and their composition showed that major phyla were Proteobacteria (mean relative abundance 0.359 ± 0.059 SD), Bacteroidetes (0.203 ± 0.030 SD), Chloroflexi (0.109 ± 0.050 SD), Actinobacteria (0.069 ± 0.014 SD), Acidobacteria (0.061 ± 0.023 SD), Verrucomicrobia (0.061 ± 0.021 SD), Patescibacteria (0.031 ± 0.014 SD), Firmicutes (0.028 ± 0.037 SD), Epsilonbacteraeota (0.027 ± 0.032 SD), and BRC1 (0.014 ± 0.006 SD) (Fig 3A, S2 Dataset). At the phylum level, members of the microbiota were the same in different tanks and at different times. The alpha diversity of the bacterial community did not show any significant differences between the tanks (Fig 3B) (Student’s t-test, *p* > 0.05). In contrast, PCoA based on the weighted UniFrac distance revealed that the bacterial community structure was strongly influenced by temporal variations, as samples from the same period clustered closely (Fig 3C). This seasonal shift was statistically supported by the environmental vector analysis (*envfit*), which identified temperature as a significant driver (*p* < 0.05) correlated with the ordination axes. However, even within this dominant temporal framework, the bacterial communities in the anaerobic/anoxic and aerobic tanks were different (anaerobic vs. aerobic: *p* < 0.005, F = 3.46, R^2^ = 0.12; anoxic vs. aerobic: *p* < 0.05, F = 2.45, R^2^ = 0.12, PERMANOVA).

Regarding the correlations between the chemical ion concentrations in the wastewater treatment process, there were positive correlations between sulfate (SO_4_^2-^), sodium (Na^+^), ammonium (NH_4_^+^), potassium (K^+^), and magnesium (Mg^2+^) in the anaerobic and anoxic tanks (Fig 2B). Na^+^, K^+^, and Mg^2+^ were also positively correlated in the aerobic tank. Temperature was negatively correlated with SO_4_^2-^ in all tanks and negatively correlated with NO_3_^-^ in the aerobic tank. As the temperature decreased, the inflow of NH_4_^+^ into the wastewater treatment plant increased; however, the activity of nitrifying bacteria may have decreased as NO_3_^-^ production also decreased. Other WWTPs reported a similar decrease in nitrification performance during the cold winter season and NH_4_^+^ amount increased [25–27].

**Fig 4:**
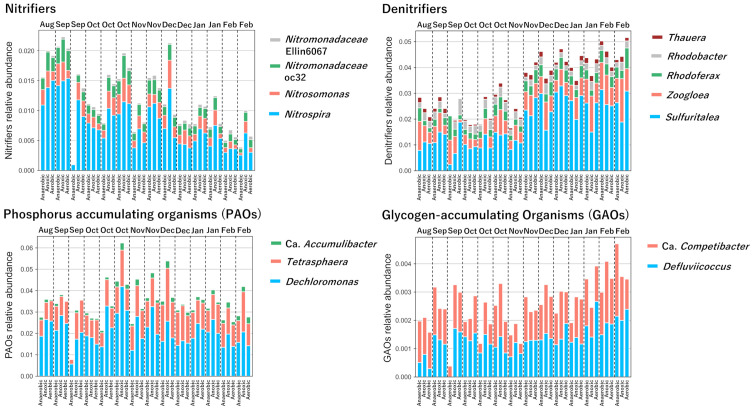
The functional guilds in A_2_O treatment process.

The functional guilds of the WWTP, categorized according to Dueholm et al. [28], are shown in Fig 4. A key observation was the decline in the relative abundance of *Nitrospira*, a primary nitrite-oxidizing bacterium, during colder months, despite the increase in NO_3_^-^ concentrations in the aerobic tank. This apparent discrepancy implies that the nitrification process was limited by substrate availability rather than bacterial abundance. As indicated by the high ammonium removal efficiency (98.4 ± 5.8%), the nitrification process was substrate limited. This means the nitrifying community, even with reduced relative abundance in winter, retained sufficient capacity to completely oxidize the influent ammonium. Therefore, the observed increase in NO_3_^−^ concentration was not due to increased bacterial activity per se but was a stoichiometric result of the increased influent NH_4_^+^ load during winter being fully converted to nitrate.

To assess whether factors other than temperature influenced the community and nitrification, we analyzed ancillary variables including ORP, MLSS, and DO (S2 Table). Throughout the study period, DO concentrations in the aerobic tank were generally maintained above 1.8 mg/L, indicating that oxygen availability was not a limiting factor for nitrification. Interestingly, MLSS concentrations increased from summer (1,340 mg/L in August) to winter (1,910 mg/L in February). This suggests an operational strategy to compensate for reduced bacterial activity in colder temperatures by increasing the total biomass. However, despite this increase in biomass, the relative abundance of *Nitrospira* decreased, and nitrate production dynamics shifted. This reinforces the conclusion that temperature-dependent reaction kinetics, rather than biomass quantity or oxygen limitation, were the primary drivers of the observed seasonal changes.

Conversely, distinct dynamics were observed in the denitrifying community. *Sulfuritalea*, a genus capable of denitrification, increased in relative abundance as temperatures dropped (Fig 4). Consistent with its reported prevalence in cold environments [29,30], this suggests a compensatory ecological response where *Sulfuritalea* proliferated to fill the niche of cold-sensitive heterotrophic denitrifiers. However, the net accumulation of NO_3_^−^ indicates that despite this compensatory shift, the overall denitrification capacity was insufficient to remove the increased nitrate generated from the high winter ammonium load. Thus, the winter condition was characterized by complete nitrification driven by high influent load, coupled with kinetically limited denitrification.

### Spatiotemporal analysis of bacterial communities in A_2_O treatment process

**Fig 5:**
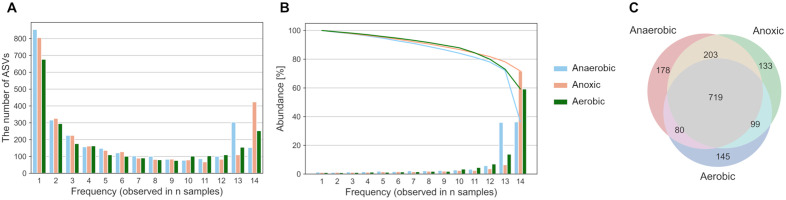
Transient and core bacteria in A_2_O treatment process. **(A)** Relationship between the number of detections and the number of ASVs in 14 time-series samplings, and (B) their bacterial abundance. **(C)** Venn diagram of ASVs detected in anaerobic, anoxic, and aerobic tanks.

To determine whether bacteria were transient or frequently detected (core) in the A_2_O treatment process, ASVs commonly detected across time points were analyzed (Fig 5A). The number of ASVs detected only once was the largest, but many ASVs were detected in common throughout the entire period, suggesting that the bacteria in the wastewater treatment plant were polarized between the transient and core. As regards the number of ASVs, frequently observed ASVs (observed in >12 of 14 samples) were the dominant community, making up >70% of the total (Fig 5B). This polarization between transient and core ASVs and the dominance of core ASVs have the same characteristics as the microbiota of the Danish wastewater treatment plant [31]. However, the bacterial ecosystem of the river was dominated by transient bacteria and not polarized between transient and core bacteria as in the WWTP (S1 Fig). The number of ASVs detected in all the three tanks was 719 (46.0%), as shown in Fig 5C. Relative abundances of these common ASVs reached 0.834 ± 0.121 SD in the anaerobic tank, 0.842 ± 0.133 SD in the anoxic tank, and 0.867 ± 0.124 SD in the aerobic tank. Consequently, the majority of core bacteria frequently detected over time were consistently detected in all three tanks, indicating a continuity in the temporal and spatial core bacteria. The stability of this dominant core community has significant implications for environmental health risks. Recent studies suggest that stable core taxa in WWTPs can serve as persistent reservoirs for antibiotic resistance genes (ARGs), facilitating their propagation regardless of transient influent fluctuations [32–34]. The polarization observed in this study, where a stable core dominates despite seasonal changes, suggests that if these core taxa harbor ARGs, the WWTP could act as a continuous source of resistomes released into the receiving river, necessitating long-term monitoring beyond standard water quality indices.

### Impact of treated wastewater on riverine bacterial communities

**Fig 6:**
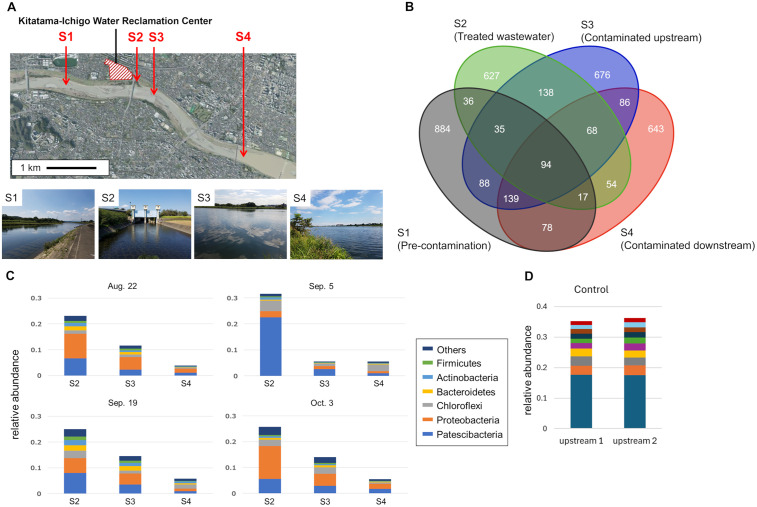
Bacterial community characteristics before and after treated wastewater discharge. **(A)** Site S1 is located before the treated wastewater discharge, site S2 is at the discharge point, and sites S3 and S4 are immediately after the discharge and 2 km downstream, respectively. The map was created by editing GSI Maps published by the Geospatial Information Authority of Japan [35] (https://maps.gsi.go.jp/#15/35.651333/139.507871/&base=ort&ls=ort&disp=1&vs=c1g1j0h0k0l0u0t0z0r0s0m0f1&d=m) under a CC BY license, original copyright 2025. **(B)** Venn diagram of ASVs detected at the four sites. **(C)** Total relative abundance of bacterial ASVs detected at sites S2 and S3 but not at site S1. The underlying data for these relative abundances are provided in S3 Dataset. **(D)** Comparison of the relative abundance of the top 10 dominant ASVs between two upstream reference sites (upstream 1 is located approximately 8.5 km upstream of upstream 2) using consistent color coding for each ASV to demonstrate natural background stability.

Wastewater is discharged into the rivers after nutrient removal and chlorine disinfection. The impact of bacterial DNA, in treated wastewater, on a river was evaluated using 16S rRNA gene analysis of river water upstream and downstream of the treated wastewater discharge (Fig 6, S3 Dataset). We identified 206 ASVs derived from bacterial DNA introduced into the river after the discharge of treated wastewater (detected at sites S2 and S3, but not at site S1) (Fig 6B). The changes in relative abundance of bacterial DNA at different sites are shown in Fig 6C. Relative abundances of the ASVs were 0.264 ± 0.037 SD at site S2, 0.114 ± 0.041 SD at site S3, and 0.052 ± 0.009 SD at site S4, indicating a significant difference in the relative abundance between sites S2 and S4 (*p* < 0.05, Friedman test; S2 vs S3: *p* < 0.05, Nemenyi post-hoc test). These results suggest that the abundance of wastewater-derived bacteria decreased significantly as the water flowed downstream. Since there are no tributary inflows between sites S3 and S4, this reduction cannot be attributed to hydrological dilution from increased water volume. Instead, it is likely caused by physical dispersion (transitioning from incomplete mixing at S3 to complete mixing at S4) and removal processes. Notably, since the wastewater is treated with sodium hypochlorite before discharge, many detected sequences likely represent DNA from inactivated bacteria (relic DNA). Thus, the downstream decline reflects not only sedimentation but also the degradation of this DNA in the natural river environment.

To confirm whether the observed changes were due to the WWTP discharge or natural spatial variation, we compared bacterial communities between two upstream reference sites separated by 8.5 km (Fig 6D). The bacterial composition in the natural upstream river environment was highly stable; the total relative abundance of the top 10 dominant ASVs showed negligible change, accounting for 35.2% at upstream 1 and 36.2% at upstream 2. In contrast, the bacterial composition changed drastically at the WWTP discharge point (Site S3) compared to the upstream site (Site S1). This stability in the upstream reference sites confirms that the community shift observed at Site S3 is a direct consequence of the treated wastewater discharge rather than natural fluctuations.

In this study, the abundance of wastewater-derived bacteria significantly decreased 2 km downstream of the treated-water discharge point, indicating that the influence of the effluent was spatially limited. This phenomenon was also observed in other rivers. Both the Riera de Cànove in Catalonia, Spain, and the Jialu in Zhengzhou City, China, exhibited a significant reduction in wastewater-associated bacteria 1 km downstream from the point of treated wastewater discharge [36,37]. In contrast, untreated wastewater discharged into a river does not lead to such mitigation, even 1 km downstream [38]. This implies that the rapid attenuation of wastewater impact observed in our study can be attributed to proper treatment of wastewater using the A_2_O process. The WWTPs in Tokyo disinfect wastewater with chlorine before discharge, except during heavy rain overflows. In addition to disinfection, it may be necessary to remove microbial DNA from wastewater. Wastewater contains a variety of antibiotic resistance genes, raising concerns that horizontal gene transfer could lead to the emergence of antibiotic-resistant bacteria [39–42]. In the present study, we observed a decrease in microbial DNA in the treated water as it flowed downstream. However, previous studies have reported that the impact of treated water persists more in river sediments than in river water [36]. Therefore, it is necessary to investigate the influence on river sediment in future studies.

## Conclusions

This study investigated the bacterial dynamics of a wastewater treatment process and its receiving river to assess the effectiveness and ecological impact of wastewater treatment. The A_2_O process effectively removed nutrients, although seasonal temperature changes influenced the nitrification. Decreased temperatures correlated with a decrease in *Nitrospira* abundance and potentially reduced nitrate production, highlighting the temperature sensitivity of the treatment process. The bacterial community in the wastewater treatment plant was polarized, with a significant portion of core bacteria consistently present over time and across different tanks, indicating a stable and functional bacterial ecosystem. The bacterial DNA introduced through the discharge of treated wastewater into the Tama River decreased as it flowed downstream, indicating dilution and dispersal. Understanding wastewater treatment processes using bacteria and their impact on riverine ecosystems is crucial for environmental conservation and preservation of water quality.

## Supporting information

S1 Table Tank capacity and residence time of each tank. (XLSX)

S2 Table Operational parameters of the A_2_O process during the sampling period. Representative monthly values for Oxidation-Reduction Potential (ORP) in anaerobic and anoxic tanks, Mixed Liquor Suspended Solids (MLSS) and Dissolved Oxygen (DO) in the aerobic tank, and influent temperature.
(XLSX)

S3 Table Geographic coordinates of the sampling sites. (XLSX)

S1 Fig Transient and core bacteria in Tama river. (A) six samplings at eight sites (TR1–TR8). The map was created by editing GSI Maps published by the Geospatial Information Authority of Japan [35] (https://maps.gsi.go.jp/#11/35.629396/139.434357/&base=english&ls=english&disp=1&vs=c1g1j0h0k0l0u0t0z0r0s0m0f1&d=m) under a CC BY license, original copyright 2025. (B) Relationship between the number of detections and the number of ASVs converted to ratio in 6 time-series samplings. Series of eight bars from white to black represent sampling sites, with white indicating TR1 and black indicating TR8.
(TIF)

S1 Dataset Ion concentration data in the A_2_O process. (XLSX)

S2 Dataset Bacterial relative abundance data in the A_2_O process. (XLSX)

S3 Dataset Bacterial relative abundance data in the river. (XLSX)

## References

[1] Seitzinger S, Harrison JA, Böhlke JK, Bouwman AF, Lowrance R, Peterson B, et al. Denitrification across landscapes and waterscapes: a synthesis. Ecol Appl. 2006;16(6):2064–90. doi: 10.1890/1051-0761(2006)016[2064:dalawa]2.0.co;2 17205890

[2] Manuel J. Nutrient pollution: a persistent threat to waterways. Environ Health Perspect. 2014;122(11):A304-9. doi: 10.1289/ehp.122-A304 25360879 PMC4216153

[3] Maavara T, Parsons CT, Ridenour C, Stojanovic S, Dürr HH, Powley HR, et al. Global phosphorus retention by river damming. Proc Natl Acad Sci U S A. 2015;112(51):15603–8. doi: 10.1073/pnas.1511797112 26644553 PMC4697372

[4] Mallin MA, Cahoon LB. The Hidden Impacts of Phosphorus Pollution to Streams and Rivers. BioScience. 2020;70(4):315–29. doi: 10.1093/biosci/biaa001

[5] Hamdhani H, Eppehimer DE, Bogan MT. Release of treated effluent into streams: A global review of ecological impacts with a consideration of its potential use for environmental flows. Freshw Biol. 2020;65:1657–70. doi: 10.1111/fwb.13519

[6] Enns D, Cunze S, Baker NJ, Oehlmann J, Jourdan J. Flushing away the future: The effects of wastewater treatment plants on aquatic invertebrates. Water Res. 2023;243:120388. doi: 10.1016/j.watres.2023.120388 37517151

[7] Albini D, Lester L, Sanders P, Hughes J, Jackson MC. The combined effects of treated sewage discharge and land use on rivers. Glob Chang Biol. 2023;29(22):6415–22. doi: 10.1111/gcb.16934 37736004 PMC10946937

[8] Albini D, Lester L, Sanders P, Hughes JMR, Jackson MC. Early detection and environmental drivers of sewage fungus outbreaks in rivers. Ecol Sol and Evidence. 2023;4(3). doi: 10.1002/2688-8319.12277

[9] González JM, de Guzmán I, Elosegi A, Larrañaga A. Tertiary wastewater treatment combined with high dilution rates fails to eliminate impacts on receiving stream invertebrate assemblages. Sci Total Environ. 2023;859(Pt 2):160425. doi: 10.1016/j.scitotenv.2022.160425 36427726

[10] Duan H, van den Akker B, Thwaites BJ, Peng L, Herman C, Pan Y, et al. Mitigating nitrous oxide emissions at a full-scale wastewater treatment plant. Water Res. 2020;185:116196. doi: 10.1016/j.watres.2020.116196 32738601

[11] Li L, Ling Y, Wang H, Chu Z, Yan G, Li Z, et al. N2O emission in partial nitritation-anammox process. Chinese Chemical Letters. 2020;31(1):28–38. doi: 10.1016/j.cclet.2019.06.035

[12] Kampschreur MJ, Temmink H, Kleerebezem R, Jetten MSM, van Loosdrecht MCM. Nitrous oxide emission during wastewater treatment. Water Res. 2009;43(17):4093–103. doi: 10.1016/j.watres.2009.03.001 19666183

[13] Drury B, Rosi-Marshall E, Kelly JJ. Wastewater treatment effluent reduces the abundance and diversity of benthic bacterial communities in urban and suburban rivers. Appl Environ Microbiol. 2013;79(6):1897–905. doi: 10.1128/AEM.03527-12 23315724 PMC3592216

[14] Kodera SM, Sharma A, Martino C, Dsouza M, Grippo M, Lutz HL, et al. Microbiome response in an urban river system is dominated by seasonality over wastewater treatment upgrades. Environ Microbiome. 2023;18(1):10. doi: 10.1186/s40793-023-00470-4 36805022 PMC9938989

[15] Lee J, Ju F, Beck K, Bürgmann H. Differential effects of wastewater treatment plant effluents on the antibiotic resistomes of diverse river habitats. ISME J. 2023;17(11):1993–2002. doi: 10.1038/s41396-023-01506-w 37684524 PMC10579368

[16] Higashi K, Suzuki S, Kurosawa S, Mori H, Kurokawa K. Latent environment allocation of microbial community data. PLoS Comput Biol. 2018;14(6):e1006143. doi: 10.1371/journal.pcbi.1006143 29874232 PMC6005635

[17] Nishida A, Nakagawa M, Yamamura M. Determinism of microbial community assembly by drastic environmental change. PLoS One. 2021;16(12):e0260591. doi: 10.1371/journal.pone.0260591 34855810 PMC8638896

[18] Nishida A, Thiel V, Nakagawa M, Ayukawa S, Yamamura M. Effect of light wavelength on hot spring microbial mat biodiversity. PLoS One. 2018;13(1):e0191650. doi: 10.1371/journal.pone.0191650 29381713 PMC5790269

[19] Bolyen E, Rideout JR, Dillon MR, Bokulich NA, Abnet CC, Al-Ghalith GA, et al. Reproducible, interactive, scalable and extensible microbiome data science using QIIME 2. Nat Biotechnol. 2019;37(8):852–7. doi: 10.1038/s41587-019-0209-9 31341288 PMC7015180

[20] Quast C, Pruesse E, Yilmaz P, Gerken J, Schweer T, Yarza P, et al. The SILVA ribosomal RNA gene database project: improved data processing and web-based tools. Nucleic Acids Res. 2013;41(Database issue):D590-6. doi: 10.1093/nar/gks1219 23193283 PMC3531112

[21] Oksanen J, Simpson GL, Blanchet FG, Kindt R, Legendre P, Minchin PR. Vegan: Community Ecology Package. 2025. doi: 10.32614/CRAN.package.vegan

[22] R Core Team. R: A language and environment for statistical computing. In: R Foundation for Statistical Computing, Vienna, Austria. URL https://www.R-project.org/

[23] Virtanen P, Gommers R, Oliphant TE, Haberland M, Reddy T, Cournapeau D, et al. SciPy 1.0: fundamental algorithms for scientific computing in Python. Nat Methods. 2020;17(3):261–72. doi: 10.1038/s41592-019-0686-2 32015543 PMC7056644

[24] Terpilowski M. scikit-posthocs: Pairwise multiple comparison tests in Python. JOSS. 2019;4(36):1169. doi: 10.21105/joss.01169

[25] Johnston J, LaPara T, Behrens S. Composition and Dynamics of the Activated Sludge Microbiome during Seasonal Nitrification Failure. Sci Rep. 2019;9(1):4565. doi: 10.1038/s41598-019-40872-4 30872659 PMC6418219

[26] Johnston J, Du Z, Behrens S. Ammonia-Oxidizing Bacteria Maintain Abundance but Lower amoA-Gene Expression during Cold Temperature Nitrification Failure in a Full-Scale Municipal Wastewater Treatment Plant. Microbiol Spectr. 2023;11(2):e0257122. doi: 10.1128/spectrum.02571-22 36786623 PMC10100873

[27] Gnida A, Wiszniowski J, Felis E, Sikora J, Surmacz-Górska J, Miksch K. The effect of temperature on the efficiency of industrial wastewater nitrification and its (geno)toxicity. Archives of Environmental Protection. 2016;42(1):27–34. doi: 10.1515/aep-2016-0003

[28] Dueholm MKD, Nierychlo M, Andersen KS, Rudkjøbing V, Knutsson S, MiDAS Global Consortium, et al. MiDAS 4: A global catalogue of full-length 16S rRNA gene sequences and taxonomy for studies of bacterial communities in wastewater treatment plants. Nat Commun. 2022;13(1):1908. doi: 10.1038/s41467-022-29438-7 35393411 PMC8989995

[29] de Almeida Fernandes L, Pereira AD, Leal CD, Davenport R, Werner D, Filho CRM, et al. Effect of temperature on microbial diversity and nitrogen removal performance of an anammox reactor treating anaerobically pretreated municipal wastewater. Bioresour Technol. 2018;258:208–19. doi: 10.1016/j.biortech.2018.02.083 29525596

[30] Zhu Y, Liu Y, Chang H, Yang H, Zhang W, Zhang Y, et al. Deciphering the microbial community structures and functions of wastewater treatment at high-altitude area. Front Bioeng Biotechnol. 2023;11:1107633. doi: 10.3389/fbioe.2023.1107633 36923457 PMC10009103

[31] Saunders AM, Albertsen M, Vollertsen J, Nielsen PH. The activated sludge ecosystem contains a core community of abundant organisms. ISME J. 2016;10(1):11–20. doi: 10.1038/ismej.2015.117 26262816 PMC4681854

[32] Zhu C, Wu L, Ning D, Tian R, Gao S, Zhang B, et al. Global diversity and distribution of antibiotic resistance genes in human wastewater treatment systems. Nat Commun. 2025;16(1):4006. doi: 10.1038/s41467-025-59019-3 40301344 PMC12041579

[33] de Nies L, Busi SB, Kunath BJ, May P, Wilmes P. Mobilome-driven segregation of the resistome in biological wastewater treatment. Elife. 2022;11:e81196. doi: 10.7554/eLife.81196 36111782 PMC9643006

[34] Raza S, Shin H, Hur H-G, Unno T. Higher abundance of core antimicrobial resistant genes in effluent from wastewater treatment plants. Water Res. 2022;208:117882. doi: 10.1016/j.watres.2021.117882 34837814

[35] Geospatial Information Authority of Japan. GSI Maps. 2025. In: https://www.gsi.go.jp/ENGLISH/index.html

[36] Lu Q, Mao J, Xia H, Song S, Chen W, Zhao D. Effect of wastewater treatment plant discharge on the bacterial community in a receiving river. Ecotoxicol Environ Saf. 2022;239:113641. doi: 10.1016/j.ecoenv.2022.113641 35597140

[37] Pascual-Benito M, Ballesté E, Monleón-Getino T, Urmeneta J, Blanch AR, García-Aljaro C, et al. Impact of treated sewage effluent on the bacterial community composition in an intermittent mediterranean stream. Environ Pollut. 2020;266(Pt 1):115254. doi: 10.1016/j.envpol.2020.115254 32721842

[38] Xie Y, Liu X, Wei H, Chen X, Gong N, Ahmad S, et al. Insight into impact of sewage discharge on microbial dynamics and pathogenicity in river ecosystem. Sci Rep. 2022;12(1):6894. doi: 10.1038/s41598-022-09579-x 35477966 PMC9044725

[39] Morina JC, Franklin RB. Drivers of Antibiotic Resistance Gene Abundance in an Urban River. Antibiotics (Basel). 2023;12(8):1270. doi: 10.3390/antibiotics12081270 37627690 PMC10451346

[40] Haenelt S, Richnow H-H, Müller JA, Musat N. Antibiotic resistance indicator genes in biofilm and planktonic microbial communities after wastewater discharge. Front Microbiol. 2023;14:1252870. doi: 10.3389/fmicb.2023.1252870 37731921 PMC10507703

[41] Khanal S, K C S, Joshi TP, Han Z, Wang C, Maharjan J, et al. Extended-spectrum β-lactamase-producing bacteria and their resistance determinants in different wastewaters and rivers in Nepal. J Hazard Mater. 2024;473:134660. doi: 10.1016/j.jhazmat.2024.134660 38795483

[42] Varela AR, Nunes OC, Manaia CM. Quinolone resistant Aeromonas spp. as carriers and potential tracers of acquired antibiotic resistance in hospital and municipal wastewater. Sci Total Environ. 2016;542(Pt A):665–71. doi: 10.1016/j.scitotenv.2015.10.124 26546762

