# Supplementary figures and images for: The role of bacteria in wastewater treatment and the impact of treated wastewater on riverine bacterial ecosystems
Source: PLoS One. 2026 Apr 15;21(4):e0346342. doi: 10.1371/journal.pone.0346342 (PMC13082631; doi:10.1371/journal.pone.0346342)

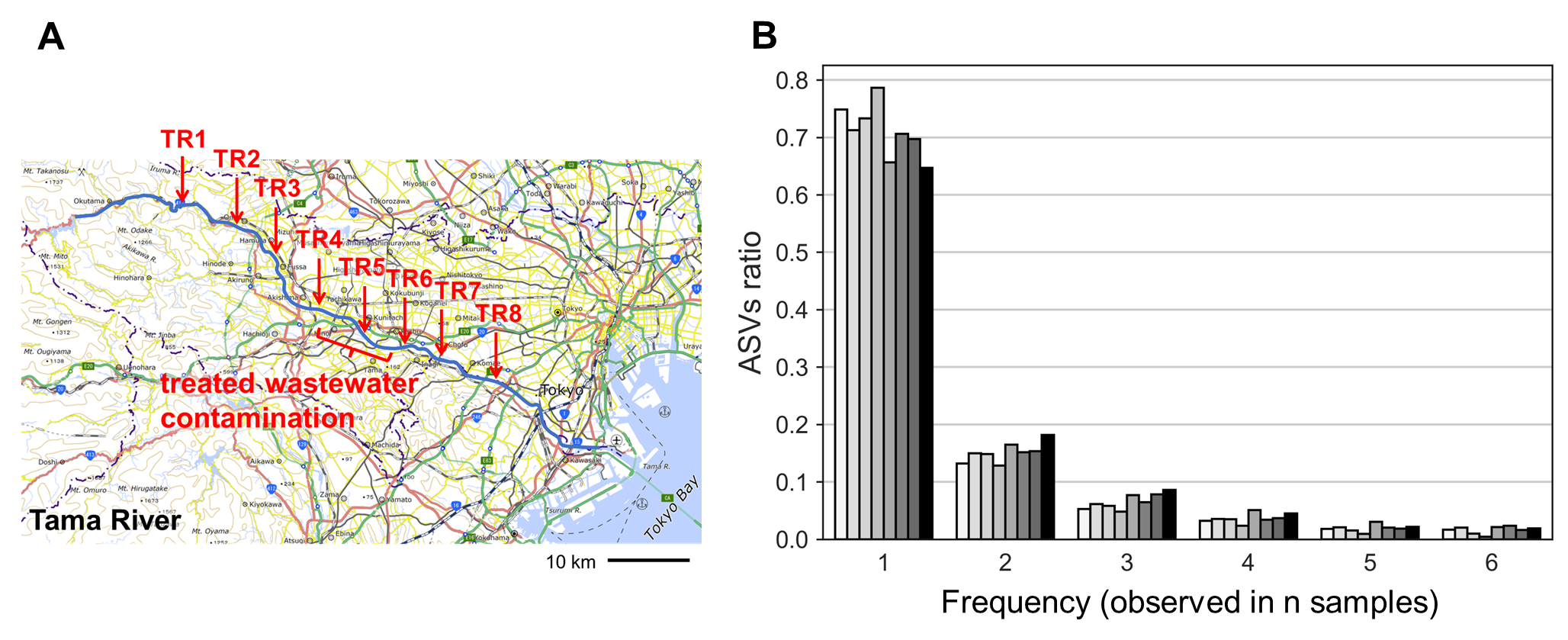

Supplement: S1 Fig — (A) six samplings at eight sites (TR1–TR8). The map was created by editing GSI Maps published by the Geospatial Information Authority of Japan [35] (https://maps.gsi.go.jp/#11/35.629396/139.434357/&base=english&ls=english&disp=1&vs=c1g1j0h0k0l0u0t0z0r0s0m0f1&d=m) under a CC BY license, original copyright 2025. (B) Relationship between the number of detections and the number of ASVs converted to ratio in 6 time-series samplings. Series of eight bars from white to black represent sampling sites, with white indicating TR1 and black indicating TR8. (TIF) [file pone.0346342.s004.tif]
