# Supplementary material for: The role of bacteria in wastewater treatment and the impact of treated wastewater on riverine bacterial ecosystems
Source: PLoS One. 2026 Apr 15;21(4):e0346342. doi: 10.1371/journal.pone.0346342 (PMC13082631; doi:10.1371/journal.pone.0346342)
Supplement: Attachment — Submitted filename: Reviewer comments .pdf [file pone.0346342.s008.pdf]

Manuscript Title: The role of bacteria in wastewater treatment and their impact on riverine bacterial ecosystems

Reviewer Recommendation: Major Revision

Overall Assessment

This well-designed study provides valuable insights into bacterial dynamics in an A_2O wastewater treatment plant and its impact on the Tama River ecosystem. While the experimental approach and integration of microbial/chemical data are strengths, several areas require enhancement to clarify scientific foundations, methodological transparency, and environmental implications before publication.*

Major Revisions Required

1. **The Introduction should explicitly state the central hypothesis** rather than implying objectives (e.g., lines 70-74), with a concise formulation such as: *"We hypothesized that seasonal temperature variations would reduce nitrifier activity in the A_2O process,……"* to frame the study's scientific intent and connect it to broader ecological risks like antibiotic resistance gene dispersal.
2. **Statistical methodologies need expanded justification**, particularly the selection of the use of Friedman/Nemenyi tests for riverine data without explaining non-parametric choices); a dedicated "Statistical Analysis" subsection should clarify software parameters, permutation counts for PERMANOVA, and p-value thresholds while justifying similarity metrics
3. **Environmental context must be strengthened** by integrating ancillary variables (e.g., pH, dissolved oxygen, organic load) into multivariate models like PCoA Fig. 3B) to avoid overreliance on temperature when explaining nitrification shifts and river recovery (lines while discussing alternative drivers such as hydraulic retention time versus temperature for nitrification efficiency and sediment interactions for DNA persistence.
4. **Contradictory findings require deeper interpretation**, specifically the decline in *Nitrospira* abundance alongside increased NO_3^- production and implications of core-transient polarization; the Discussion should address microbial functional redundancy (e.g., *Sulfuritalea*'s compensatory role) and connect core bacterial stability to antibiotic resistance risks using recent literature.

Minor Revisions

1. **Figures need enhanced clarity**: Label functional guilds on y-axes in Fig. 4 and define color keys for temporal trends, add geographic coordinates and scale bars to the river map in Fig. 6A, and incorporate α-diversity statistics from S1 Fig into the main text to support tank comparisons.

2. **Language requires standardization and polishing**: Professional English editing is recommended to address grammatical errors.

3. **References need reorganization and formatting**: Sort chronologically to highlight thematic progression (e.g., group nitrification studies [22-24,26]), italicize genus/species names (e.g., *Nitrospira*), and ensure DOI consistency (reference 18 currently lacks DOI).

4. **Data accessibility should be clarified**: Explicitly state whether sediment data referenced in lines 239-240 are archived in repositories.
